# A18 EXPLORING TRAINED IMMUNITY IN INTESTINAL EPITHELIAL CELLS DURING TYPE 2 IMMUNE RESPONSES

**DOI:** 10.1093/jcag/gwae059.018

**Published:** 2025-02-10

**Authors:** Z L Levy, A Needs, M Richer, M Oudhoff

**Affiliations:** Health Science, Carleton University, Ottawa, ON, Canada; Health Science, Carleton University, Ottawa, ON, Canada; Health Science, Carleton University, Ottawa, ON, Canada; Health Science, Carleton University, Ottawa, ON, Canada

## Abstract

**Background:**

Helminth infections are a serious health threat that remain understudied for how frequently they occur globally. Although antihelminth drugs exist today, reinfection and drug resistance emphasize the need to understand how our body handles subsequent helminth infections, especially in the gastrointestinal system. Normally, type 2 immunity facilitates expulsion of worms from the gastrointestinal tract by relying upon a type 2 immune cell–epithelial response circuit via interleukins (IL)-25 and IL-13 signaling to upregulate helminth-sensing tuft and mucus-producing goblet cells. Recent advances in trained immunity, an evolving field in immunology where it is believed that a previous stimulus can cause changes to the epigenome, demonstrate that stimulating epithelial cells can result into better preparedness for subsequent stimuli *via* epigenetic changes.

**Aims:**

It is unknown currently whether trained immunity takes place in intestinal epithelial stem cells or how trained immunity affects intestinal stem cell differentiation during a type 2 immune response. This project aims to define whether the intestinal epithelium responds differently to a primary challenge compared to a secondary challenge during a type 2 response. In addition, we hope to identify how such memory would be orchestrated.

**Methods:**

We stimulated intestinal organoids with IL-13 to simulate a type 2 response. Organoids exposed to IL-13 display similar properties to that of the epithelium *in vivo* upon a helminth infection; i.e. tuft and goblet cell hyperplasia. Upon media replacement, organoids return to homeostasis rapidly, upon which we could re-expose them. Thus, organoids were exposed to IL-13 either once or twice and changes were analyzed by flow cytometry, immunofluorescent confocal microscopy, western blot, and bulk RNA sequencing. In the future, we will strengthen this approach by performing *in vivo* experiments.

**Results:**

We found that a secondary challenge led to an increased goblet cell response when compared to a primary one. We found that both the number of goblet cells (by flow cytometry), but more importantly, levels of goblet-specific antimicrobial protein RELM-β were strongly increased (western blot and confocal imaging). We are currently assessing the tuft cell responses to determine if it is an overall increased response to IL-13, or whether there is a preferential response (towards goblet cells instead of tuft cells).

**Conclusions:**

Our preliminary data as well as our current experiments strongly suggest that epithelium responds differently to a secondary Type 2 immune challenge compared to a primary one. Ultimately, gaining an understanding of the mechanisms that mediate intestinal epithelial-intrinsic memory can help develop new strategies for treatment of gastrointestinal infections to help reduce the burden faced by those who encounter helminth infections around the world.

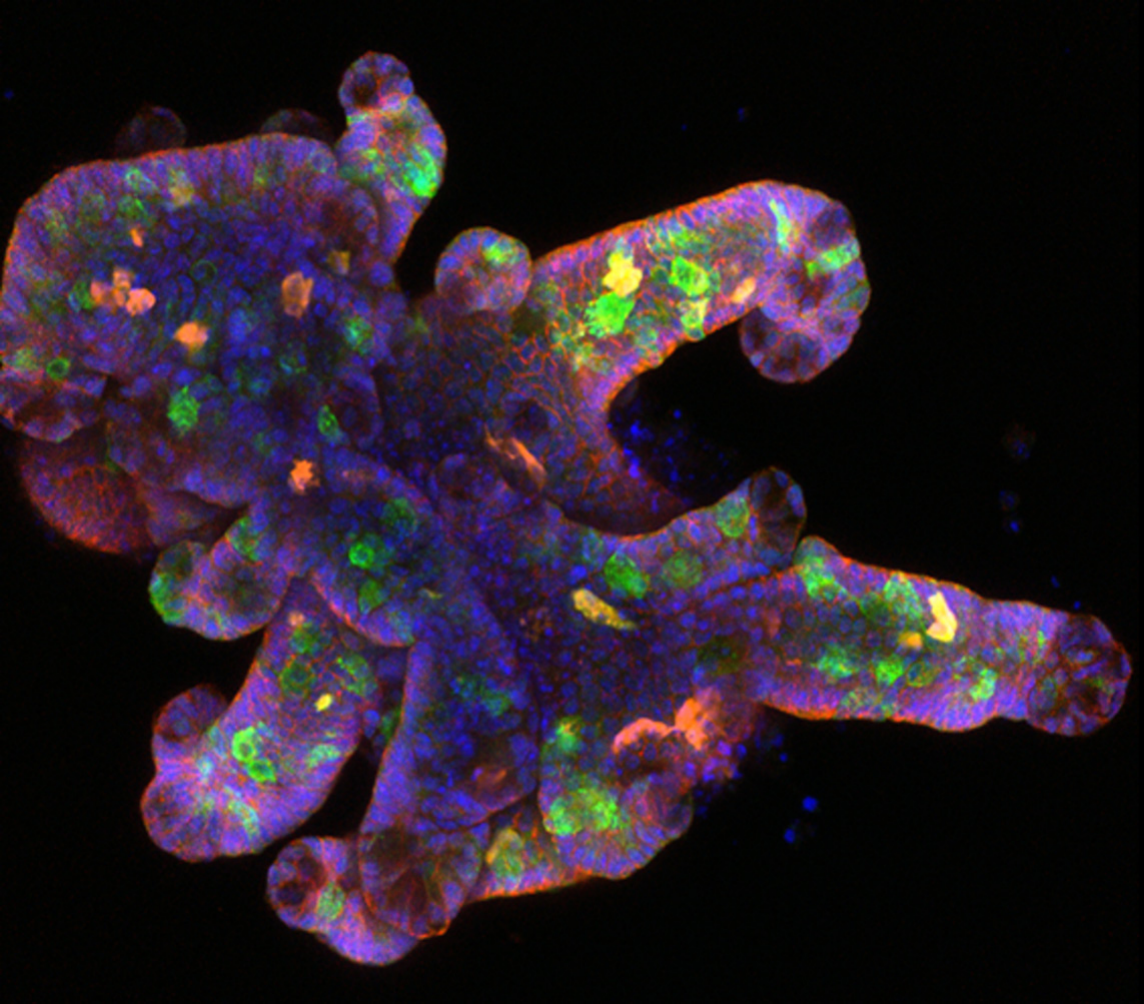

**Funding Agencies:**

CIHR

